# Interconnected Pathways: Exploring Inflammation, Pain, and Cognitive Decline in Osteoarthritis

**DOI:** 10.3390/ijms252211918

**Published:** 2024-11-06

**Authors:** Mihails Tarasovs, Sandra Skuja, Simons Svirskis, Liba Sokolovska, Andris Vikmanis, Aivars Lejnieks, Yehuda Shoenfeld, Valerija Groma

**Affiliations:** 1 Department of Internal Diseases, Riga Stradins University, Hipokrata Str. 2, LV-1038 Riga, Latvia; 2 Autoimmunity Center, Riga East University Hospital, Clinic Gailezers, Hipokrata Str. 2, LV-1038 Riga, Latvia; 3 Joint Laboratory of Electron Microscopy, Institute of Anatomy and Anthropology, Riga Stradins University, Kronvalda Blvd 9, LV-1010 Riga, Latvia; 4 Institute of Microbiology and Virology, Riga Stradins University, Ratsupites Str. 5, LV-1067 Riga, Latvia; 5 Department of Orthopaedics, Riga Stradins University, Hipokrata Str. 2, LV-1038 Riga, Latvia; 6 Zabludowicz Center for Autoimmune Diseases, Sheba Medical Center, Tel-Hashomer, Ramat Gan 52621, Israel

**Keywords:** osteoarthritis, inflammation, cognitive decline, pain, urinary biomarkers

## Abstract

The relationship among inflammation, pain, and cognitive decline in osteoarthritis (OA) patients is complex and has not been sufficiently explored; therefore, we undertook this research to evaluate how OA-related inflammation and pain affect cognitive functions, as well as to examine the potential of urinary markers as indicators of these conditions. This study examined fifty OA patients through clinical and cognitive assessments, morphological analyses, urinary biomarkers, and bioinformatics. Morphologically, 24% of patients had moderate to high synovial inflammation, which was significantly correlated with depressive symptoms, pain intensity, and self-reported anxiety. The Montreal Cognitive Assessment indicated minimal decline in most patients but showed negative correlations with age and inflammation severity. Urinary TNF-α and TGF-β1 levels positively correlated with body mass index and pain and synovitis score and immune cell infiltration, respectively. In contrast, cartilage oligomeric matrix protein and C-telopeptides of type II collagen showed inverse correlations with pain duration and cognitive function, respectively. Distinct patient clusters with higher inflammation were identified and were associated with reported pain and depressive symptoms. Urinary TNF-α and TGF-β1 can serve as biomarkers reflecting inflammation and disease severity in OA. This study suggests that synovial inflammation may be linked to mental and cognitive health in some patient cohorts.

## 1. Introduction

Osteoarthritis (OA) is a chronic, disabling disease that affects the joints and decreases locomotion ability [1,2]. As OA’s incidence and global burden grow with age, it becomes one of the leading causes of pain and disability [3]. Various genes, along with other risk factors, such as older age, female sex, overweight or obesity, previous injuries, and occupational factors like knee bending, kneeling, and squatting, as well as lifestyle factors, including sports activities like heavy lifting and alignment issues, such as varus or valgus, are recognized contributors to the onset and progression of OA [4,5,6].

Extensive observational studies using magnetic resonance imaging (MRI) and radiographs have identified various factors that contribute to the pain syndrome in OA [7]. However, some studies show no direct correlation between bone marrow edema on MRI and pain syndrome. In turn, cartilage loss seems to be slightly associated with an increase in the Western Ontario and McMaster Universities Arthritis Index (WOMAC), and this association is influenced by synovitis [8,9]. Furthermore, inflammation significantly influences pain syndrome, with evidence supporting the correlation of TNF-α, Il-1β, and IL-6 with pain syndrome in early knee OA [10]. One of the key pathological aspects of chronic pain in OA is central sensitization, which has been detected and confirmed through quantitative sensory testing analyses and functional MRI [11,12]. This indicates that the pain experienced by OA patients goes beyond the joint and involves complex neural mechanisms that contribute to chronic pain.

Patients with OA often report experiencing depression and anxiety [13]. The prevalence of depression in OA patients is sizable at 19.9, as is the prevalence of anxiety at 21.3% [14]. Both depression and OA are disabling conditions that have a significant impact on a patient’s life [15]. OA patients with depressive disorders experienced more functional disability before endoprosthetic surgery and had lower rates of recovery after it [16]. Other studies show that pain affects patients’ cognition and memory [17]. Cognitive decline has emerged as a crucial aspect requiring deeper investigation in the context of OA. Inflammatory arthritides have shown a correlation with cognitive decline, as both conditions involve cytokine-driven processes and painful manifestations [18,19,20]. While some evidence suggests low systemic inflammation in OA [21,22], it is important to recognize that even slight systemic inflammation may have far-reaching consequences, possibly affecting the central nervous system and leading to cognitive decline, depression, and anxiety. Understanding the relationship between inflammation and cognitive changes is essential in comprehending the full impact of OA on patients’ overall well-being. Research indicates that inflammation, which initiates and drives the pain syndrome in OA while also contributing to joint destruction, may also play a role in cognitive decline [23]. Some studies have demonstrated a faster reduction in brain hippocampal volume in OA patients, although their cognitive functions may not show apparent changes [24,25]. Studies on mice have provided more insights into how inflammation affects memory, revealing processes like chemokine secretion into perivascular spaces, which have been found both in patients with chronic pain and those with OA [26,27,28]. Studies exploring the impact of chronic pain, even with low-grade inflammation, have shown how it can influence patients’ overall well-being and neuropsychiatric status [29]. Thus, addressing cognitive decline in OA requires a holistic understanding of the disease, incorporating its inflammatory aspects and their effects on the central nervous system. Elucidating the mechanisms underlying cognitive decline in OA is crucial for developing targeted interventions to enhance patients’ cognitive health and improve their overall quality of life. Furthermore, the recognition of the neuropsychiatric impact of OA can lead to a more comprehensive and empathetic approach to managing this condition in affected individuals.

The breakdown of articular cartilage and chronic low-grade synovitis remain the most studied issues related to OA [30]. Synovial inflammation in the OA joint and the activation of synovial cells occur due to various factors, including mitochondrial dysfunction, damage-associated molecular patterns, cytokines, metabolites, and crystals in the synovium. The inflammatory microenvironment, in turn, leads to aberrant cartilage metabolism, contributing to further cartilage degeneration and the progression of OA [31,32,33,34,35]. The cartilage oligomeric matrix protein (COMP) and crosslinked C-telopeptides of type II collagen (CTX-II) are biochemical markers derived from the breakdown of the cartilage extracellular matrix. They have the potential to predict the early deterioration of articular cartilage and confirm the severity of OA [36,37,38,39]. Apart from serum and synovial fluid markers, urinary metabolites, including CTX-II, have demonstrated potential in predicting and monitoring disease progression in OA, indicating their promise as non-invasive biomarkers [40,41,42,43].

The aim of this study is to comprehensively investigate the interplay between inflammation, pain, and cartilage degradation biomarkers in urine and cognitive decline in OA. This involves evaluating how OA-related inflammation and pain affect cognitive functions and examining the potential of urinary markers as indicators of these conditions.

## 2. Results

### 2.1. Analysis of General Clinical Data

**Figure 1:**
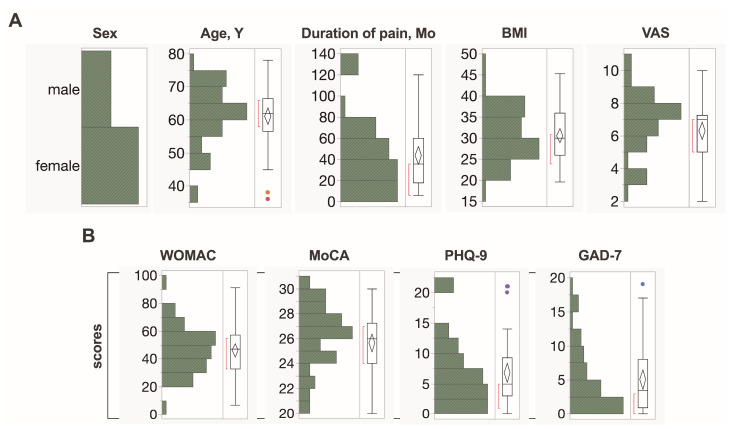
Distribution plots of the descriptive analysis. Red dots indicate lower outliers and violet dots show upper outliers. The standard box plot on the right side of the graph shows the median and the spread of values, with a red square bracket indicating the region of higher data density for the corresponding variable. (**A**) General clinical data, including the distribution of males and females in the study cohort, patients’ age in years, duration of pain in months, BMI indices, and VAS indices. (**B**) Data from standardized assessment instruments for mental and physical health and cognitive function of OA patients, including WOMAC, MoCA, PHQ-9, and GAD-7 score indices.

**Figure 2:**
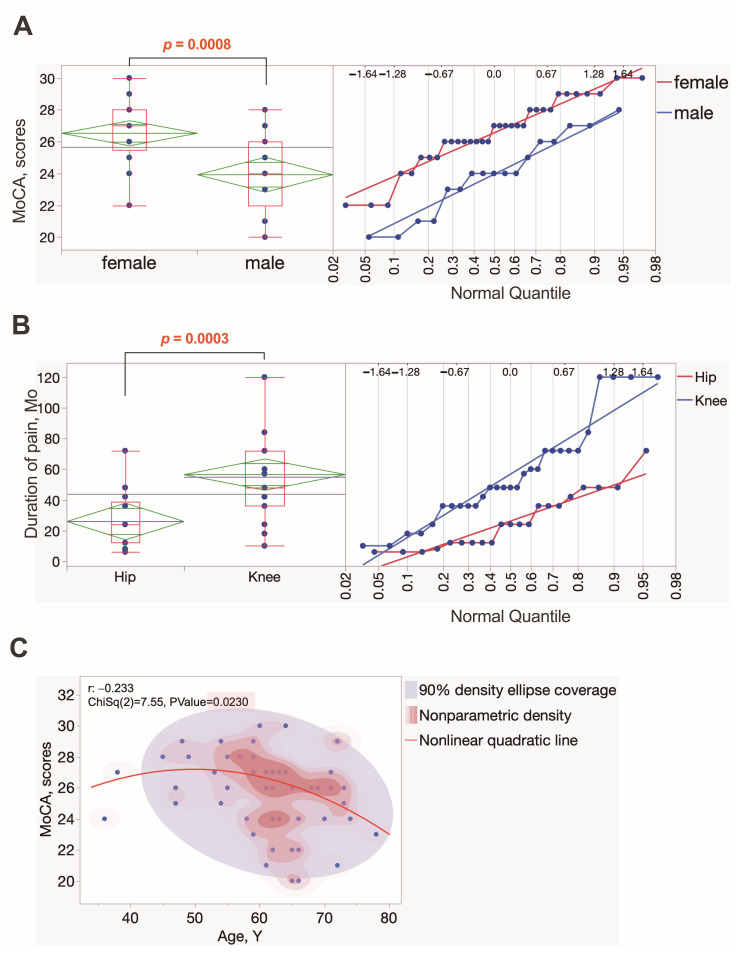
Most significant associations among general clinical data, duration of pain, and MoCA scores in OA patients. Each dot represents a single data point. (**A**) MoCA scores were significantly higher in OA females compared to males. (**B**) Difference in duration of pain between knee and hip osteoarthritis groups, showing longer symptomatic pain periods in knee OA patients within the study cohort. (**C**) A negative correlation was observed between MoCA scores and patients’ ages. The nonparametric score density plot indicates that the highest MoCA scores are predominantly observed in 55- to 75-year-old OA patients.

Among the fifty OA patients, 17 (34%) were males and 33 (66%) were females. Twenty-nine patients underwent knee replacement surgery, and 21 underwent hip endoprosthetic surgery. The median patient age was 62 (95% CI 58.5–63.7), ranging from 36 to 78 years old (Figure 1A). The median duration of pain in months was 36 (95% CI 35–52.6), ranging from 6 to 120 months. The median body mass index (BMI) was 29.97 kg/m^2^ (95% CI 28.9–32.2 kg/m^2^), ranging from 19.59 to 45.26 kg/m^2^. The mean VAS was 7 (95% CI 5.7–6.8), ranging from 2 to 10. There were no statistically significant differences between patient age, BMI, VAS, or duration of pain between the two groups of men and women. There were significant differences in pain duration time (Figure 2B) between patients with hip or knee OA (*p* = 0.003). The median duration of pain in knee OA patients was 48 months (95% CI 22.4–38.6), ranging from 10 to 120 months, whereas the median duration of pain in hip OA patients was 24 months (95% CI 12–36), ranging from 6 to 72 months. No difference in data distribution in age, BMI, or VAS between hip and knee OA was found.

### 2.2. Self-Assessment and Cognition Survey for OA Patients

The WOMAC score, which measures the severity of OA symptoms, showed a wide range of results. The median score was 47, with a range of 7 to 91, indicating significant variation in the severity of OA symptoms among the patients (Figure 1B). No differences were observed between the groups of patients according to the location of the OA and the patients’ sex.

The median MoCA score was 26 (95% CI 24.9–26.4), ranging from 20 to 30. Considering that the cut-off point for MoCA is below 26, most patients had minimal or no loss of cognitive function (Figure 1B). We found a difference in the data distribution between males and females (*p* = 0.0008). The median MoCA score among men was 24 (95% CI 22.8–26), ranging from 20 to 28, and the median score for women was 27 (95% CI 26–28), ranging from 22 to 30 (Figure 2A). Additionally, a negative correlation was found (Figure 2C) between the age of the patients and the MoCA score (r = −0.233, *p* = 0.023).

The median PHQ-9 score was 5 (95% CI 5.2–8.3), ranging from 0 to 21, indicating that the patients had mild depressive symptoms overall (Figure 1B). No correlation was found between patient age (*p* = 0.466) and duration of pain (*p* = 0.511). No statistically significant difference was observed between the sexes.

The median GAD-7 score was 3.5 (95% CI 3.6–6.5), ranging from 0 to 19, indicating that most patients had no or minimal anxiety symptoms (Figure 1B). No correlations were found between patients’ age (*p* = 0.431) or duration of pain (*p* = 0.753). No statistically significant difference was observed between the sexes.

### 2.3. Histopathological Assessment of Synovial Tissue Samples Correlated with Cognitive Survey Indices

**Figure 3:**
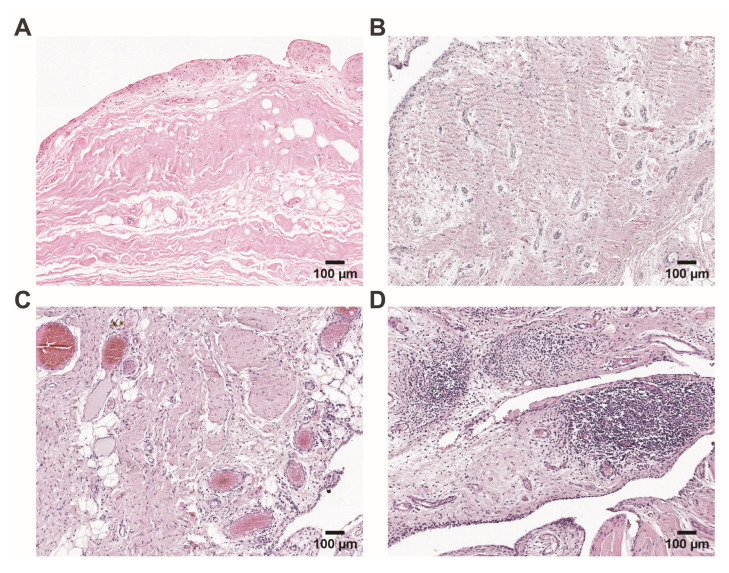
Representative histopathological images of OA depicting (**A**,**B**) fibrotic changes in otherwise non-thickened synovial lining and non-inflamed tissue; (**C**) congested blood vessels with perivascular infiltration and synovial sub-lining fibrotic changes; and (**D**) synovial inflammatory lymphocytic infiltrates. Hematoxylin and eosin staining. Scale bars: 100 μm.

All 50 patients in this study underwent a synovial biopsy during an endoprosthetic operation. A histological analysis of the synovial tissue revealed predominantly fibrotic changes (Figure 3A,B) and the presence of congested capillaries (Figure 3C), alongside occasional inflammation of the synovium (Figure 3D). Inflammation is mainly manifested as occasional inflammatory cells and lymphocytic infiltrates. Despite OA typically being considered a disease with low-grade inflammation, the analysis of immune cell infiltrates and lining and sub-lining stromal density showed significant data scattering. The median synovitis score was 3 (95% CI 2.6–4), ranging from 0 to 7. An analysis of the grade of synovial inflammation revealed that 76%, or 38, of the OA patients presented with low-grade inflammation, whereas 24%, or 12, had medium to high inflammation. No differences in synovitis score were observed between the patients’ sex (*p* = 0.168) and the localization of OA (*p* = 0.619). Additionally, no correlation was found between OA patient age and synovitis score (*p* = 0.903).

**Figure 4:**
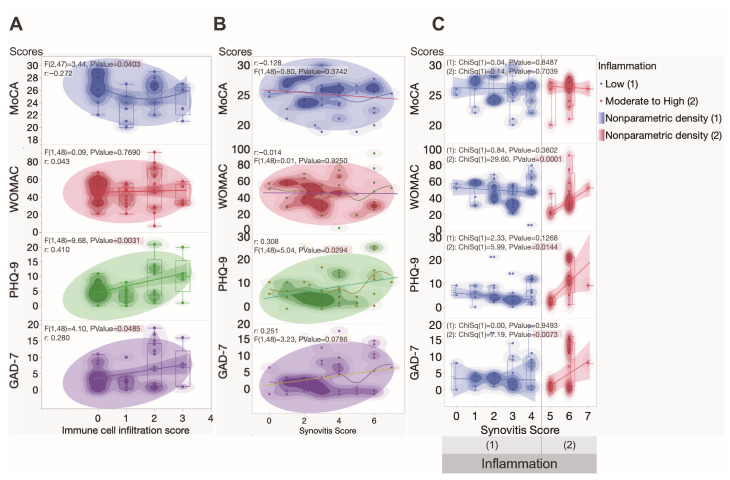
Graphs depicting correlations among self-assessment and cognition surveys for OA patients and immune cell infiltration score, synovitis score, and inflammation grade: (**A**) A significant negative correlation among immune cell infiltration and MoCA score (r = −0.272, *p* = 0.0403), PHQ-9 (r = 0.410; *p* = 0.0031), and GAD-7 (r = 0.280, *p* = 0.0485) was found. (**B**) A significant positive correlation was observed between PHQ-9 scores and synovitis scores (r = 0.304, *p* = 0.032). (**C**) Positive correlations were observed among WOMAC (*p* < 0.001, χ^2^ = 29.6), PHQ-9 (*p* = 0.0144, χ^2^ = 5.99), and GAD-7 (*p* = 0.0073, χ^2^ = 7.19) scores in the group of OA patients with a moderate to high synovial inflammation grade.

Simultaneously, significant correlations (Figure 4A) were observed between the inflammatory infiltrate grade and MoCA scores (r = −0.272, *p* = 0.0403), PHQ-9 scores (r = 0.410, *p* = 0.031), as well as GAD-7 (r = 0.280, *p* = 0.0485). These findings provide additional evidence of a potential link among inflammatory processes, depressive symptoms, and cognitive survey in OA patients.

A significant positive correlation was observed between PHQ-9 scores and synovitis scores (r = 0.308, *p* = 0.0294), indicating a potential relationship between depressive symptoms and synovial inflammation in OA patients (Figure 4B). However, no significant correlations were found among GAD-7 scores (*p* = 0.0786), WOMAC scores (*p* = 0.9250), and MoCA scores (*p* = 0.3742) with synovitis scores.

The stratification of patients based on inflammatory infiltrate grade provided additional insights. We observed a significant positive correlation between this grade and WOMAC scores in the subgroup of OA patients with moderate to high synovial inflammation, indicating a possible association between inflammation severity and the patient’s condition (χ^2^ = 29.6, *p* < 0.0001) (Figure 4C). Moreover, this subgroup demonstrated significant positive correlations with both PHQ-9 (χ^2^ = 5.99, *p* = 0.0144) and GAD-7 scores (χ^2^ = 7.19, *p* = 0.00373). No significant correlation was found with MoCA scores in this subgroup. Notably, patients in the low-synovial inflammation group exhibited no significant correlations with the assessed parameters.

Additional analysis found a positive correlation between WOMAC indices and PHQ-9 (r = 0.409, *p* = 0.003), as well as between GAD-7 (r = 0.405, *p* = 0.003). No correlation between WOMAC and MoCA has been found.

**Figure 5:**
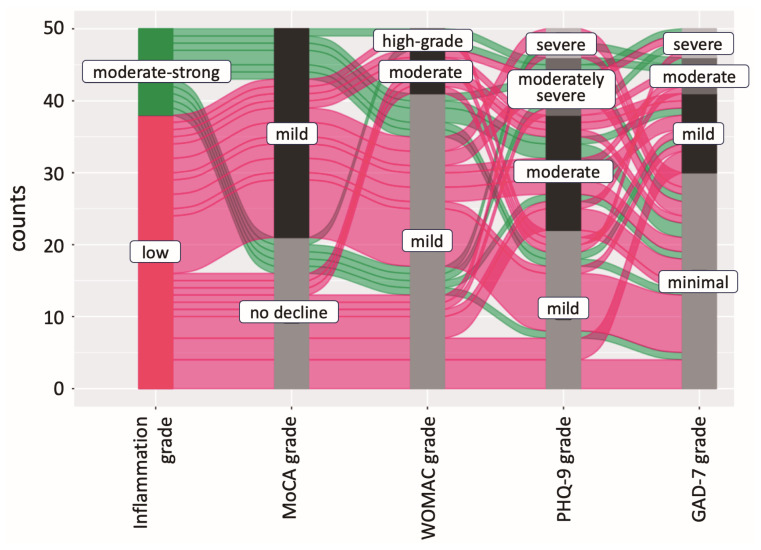
Alluvial diagram representing flows among nodes, with individual OA patient indices shown as rows and inflammation grade, patient-reported outcomes, and cognitive survey data shown as columns. The color gradient of the flow demonstrates varying degrees of inflammation, with red symbolizing a low inflammation grade and green indicating a moderate to severe grade. The thickness of each line and the flow it illustrates are based on the relative proportion of each category’s total. The plot indicates that most OA patients experienced mild cognitive changes, while a significant proportion of those with moderate- to high-grade inflammation exhibited severe depressive and anxiety symptoms, with some also displaying moderate levels of these symptoms.

Additionally, alluvial diagrams were created to illustrate the distribution of associations among the categorical dimensions of the variables. This type of data visualization was used to improve clarity in representing the distinct range of variables associated with the study (Figure 5).

### 2.4. Analysis of Urinary CTX-II, COMP, TNF-α, and TGF-β1 Enzyme-Linked Immunosorbent Assay Data

After analyzing urine samples from 21 out of 50 patients for CTXII, COMP, and TNF-α, respectively, TGF-β1 was assessed for 13 patients. The median concentration of urine CTXII was 233.13 pg/mL (ranging from 111.7 pg/mL up to 641.88 pg/mL, with a standard deviation of 142.6). Urinary COMP ranged from 12.36 ng/mL to 19.4 ng/mL, with a median value of 14 ng/mL (standard deviation 1.7). The median level of urinary TNF-α was 4.09 pg/mL, ranging from 3.43 to 7.12 pg/mL (standard deviation 0.87). Finally, urinary TGF-β1 median levels were 26.1 pg/mL, with a range from 1.46 up to 296, showing considerable data scattering.

Regarding urinary TNF-α, a positive correlation was found between patients’ BMI index (r = 0.29 with *p* = 0.029) and VAS measurements (r = 0.518 with *p* = 0.016). A positive correlation was found between synovitis score and urinary TGF-β1 (r = 0.601, *p* = 0.03). Additionally, TGF-β1 showed a positive correlation with immune cell infiltration, which is a component of the combined synovitis score (r = 0.634, *p* < 0.02). No correlation between inflammation and urinary CTXII was found. However, a negative correlation with MoCA was observed (r = −0.63, *p* = 0.002). Urinary COMP showed a negative correlation with the duration of pain (r = −0.448, *p* = 0.42); furthermore, a negative correlation was found between urinary COMP and the VAS measurements (−0.546, *p* = 0.01). No correlation between VAS and duration of pain was observed, suggesting COMP has independent correlations with these two values.

**Figure 6:**
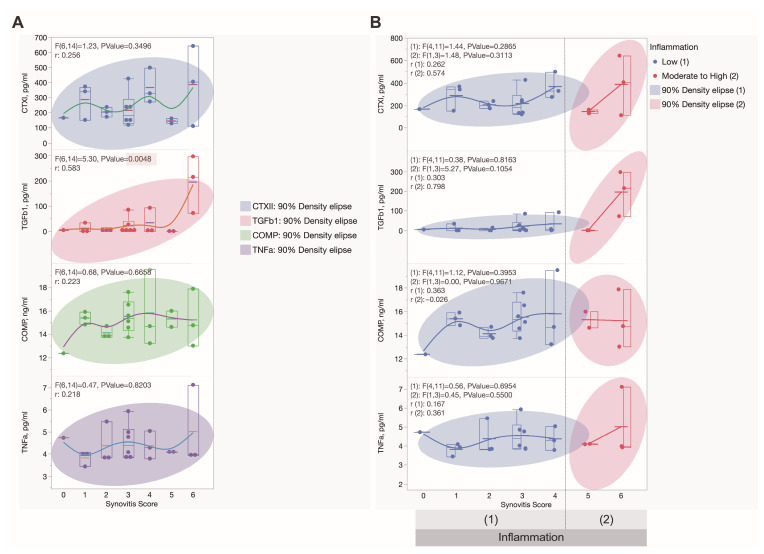
Graphs representing correlations among urinary CTX-II, COMP, TNF-α, and TGF-β1 ELISA measurements for OA patients and synovitis score and inflammation grade: (**A**) A statistically significant correlation was observed between TGF-β1 levels and synovitis score. (**B**) No statistically significant correlations were observed between urinary ELISA measurements and inflammation grade.

Using the nonparametric Mann–Whitney test, a difference in data between low- and high-inflammation groups in urinary TGF-β1 patients was found. However, no differences were found in urinary TNF-α, CTXII, and COMP (Figure 6). All urine samples showed no difference between males and females. Moreover, no differences in knee or hip OA were observed. Additionally, no differences in urine samples were found at different KL stages of OA patients.

### 2.5. Characterization of Clusters of OA Study Participants

**Figure 7:**
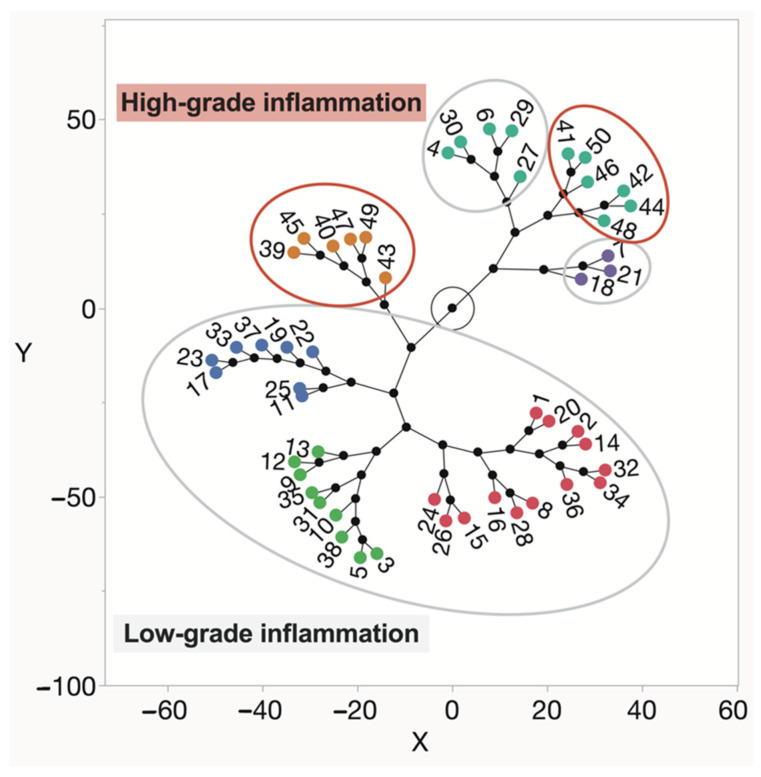
Constellation plot from the cluster analysis displays groups of OA patients with varying levels of synovial inflammation, ranging from low- to high-grade. Constellation plot colored circles represent the respective classes of Figure 8 and Figure 9.

We conducted an in-depth analysis of cell infiltration in synovial tissue due to its strong association with synovial inflammation. A cluster analysis stratified the data into several clusters based on the intensity of cell infiltration, distinguishing between low-grade and high-grade synovial inflammation (Figure 7). Further investigation revealed differences within the clusters identified in this study.

**Figure 8:**
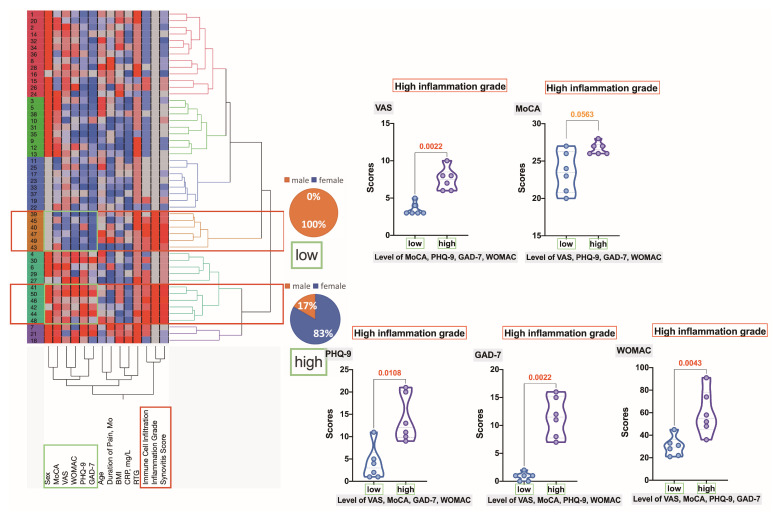
Hierarchical clustering dendrogram reveals two distinct clusters of OA patients with high-grade synovial inflammation. Variables are scaled in colors from blue to red, representing concentrations ranging from the lowest to the highest. The violin plots illustrate the differences between the factors that determined the stratification into these two subgroups. Decimal numbers indicate the exact *p*-values obtained using the Mann–Whitney test.

Within clusters exhibiting high-grade synovial inflammation (Figure 8), two distinct branches emerged: one characterized by a predominance of males with high scores in PHQ-9, GAD-7, WOMAC, and MoCA, and the other consisting of 83% females with elevated scores in PHQ-9, GAD-7, VAS, and WOMAC. Significant statistical differences were observed between these two subgroups in VAS (*p* = 0.0022), PHQ-9 (*p* = 0.0108), GAD-7 (*p* = 0.0022), and WOMAC (*p* = 0.0043); however, no significant difference was found in MoCA scores.

**Figure 9:**
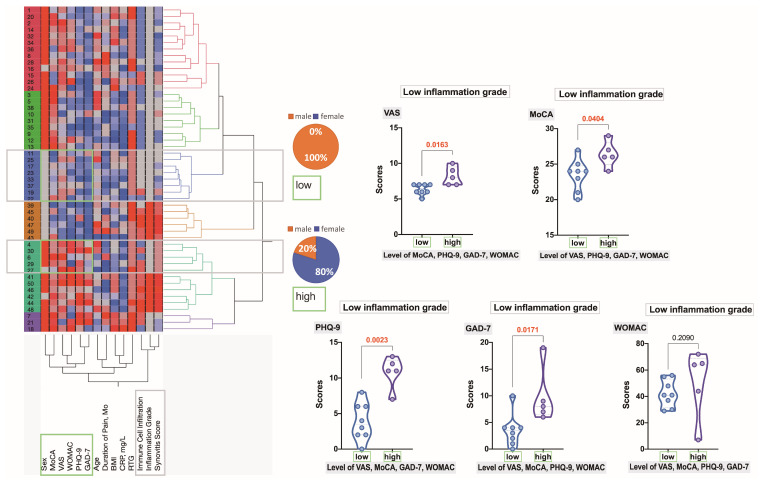
Hierarchical clustering dendrogram reveals two distinct clusters of OA patients with low-grade synovial inflammation. Variables are scaled in colors from blue to red, representing concentrations ranging from the lowest to the highest. The violin plots illustrate the differences between the factors that determined the stratification into these two subgroups. Decimal numbers indicate the exact *p*-values obtained using the Mann–Whitney test.

Conversely, another cluster displayed a scattering of data indicative of low-grade inflammation, with two distinct subgroups identified (Figure 9). One subgroup was predominantly male and showed mostly low inflammation, while the other, predominantly female (80%), exhibited higher self-reported outcomes. Significant differences between these subgroups were observed in VAS (*p* = 0.0163), MoCA (*p* = 0.0404), PHQ-9 (*p* = 0.0023), and GAD-7 (*p* = 0.0171). However, no significant difference was found in WOMAC scores (*p* = 0.2090).

**Figure 10:**
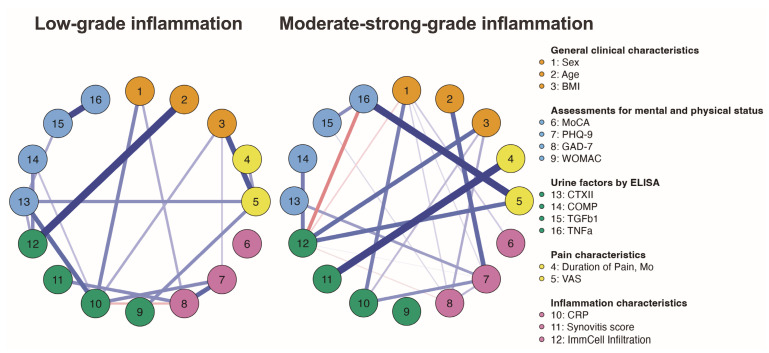
Comparative Bayesian correlation networks, based on the grade of inflammation, were constructed for five variable groups: general clinical data, mental and physical status assessments, pain characteristics, CRP levels, and morphologically confirmed synovial inflammation. Blue lines indicate positive associations, while red lines indicate negative associations.

Comparing the two clusters with high and low inflammation, both of which had a predominance of males, we found statistically significant differences only in GAD-7 scores (*p* = 0.02). Finally, a compact and interpretable representation of a joint probability distribution—a Bayesian network—was constructed (Figure 10). This tool aimed to discover and represent causal relationships between variables.

### 2.6. Ultrastructural Alterations of the Synovial Membrane and Collagen Synthesis Confirmed Using Transmission Electron Microscopy

**Figure 11:**
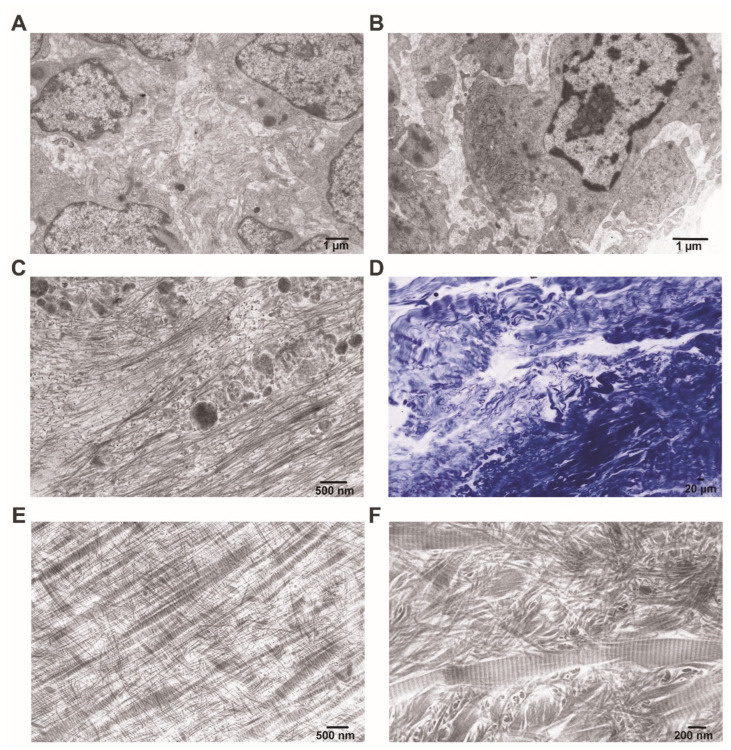
Transmission electron micrographs of representative synovial images obtained from OA patient tissue samples. (**A**,**B**) Demonstrate architectural peculiarities of the synovial cellular content. (**C**) Depicts the presence of cell organelles and their remnants along with various vacuoles localized between collagen microfibrils. (**D**–**F**) Collagen fibrils reveal diverse orientations, often cross-stitched or interwoven (**E**), and varying thickness (**F**). Only the thicker fibrils display a regular banding pattern. (**D**) Semithin section stained with 1% toluidine blue. Scale bars: (**A**,**B**) 1 µm; (**D**) 20 µm; (**C**,**E**) 500 nm; (**F**) 200 nm.

Typical synovial lining cells obtained from an OA patient with low-grade inflammation, as observed under light microscopy, were irregularly shaped and exhibited cytoplasmic processes that often appeared to overlap and varied greatly in thickness and length. Some of these processes extended secondary projections before terminating, and some contained micropinocytotic vesicles, with vesicular membranes continuous with the cell membrane (Figure 11A,B). The lining cells were separated by relatively narrow intercellular spaces, with some cells closely apposed, forming primitive contacts. A finely granular material was abundant in the intercellular spaces between the cytoplasmic processes. Additionally, abundant vacuoles containing granular and dense material—suggesting an active interchange with the extracellular matrix—along with cell remnants interspersed with collagen microfibrils, were observed (Figure 11C). The nuclei of these cells were often oval-shaped, although many exhibited irregularities in the nuclear envelope. The cellular interior was characterized mainly by abundant ergastoplasm, and some cells contained granules and vacuoles filled with dense material. Autophagosome formation with circumferentially arranged membranes of the smooth endoplasmic reticulum was also observed. In OA patients, the ultrastructural features characteristic of a more aggressive myofibroblast-like phenotype were not observed. Notably, OA patients with a moderate to high grade of inflammation were presented with lymphocytes, some plasma cells, and macrophages located at the lining and sub-lining interface, as well as perivascularly. Given the importance of characterizing collagen fibrils to monitor changes in OA, a detailed assessment of the extracellular matrix was performed. Fibrotic changes were observed in the sub-lining of the OA synovial samples, which were confirmed at both the light (Figure 11D) and electron microscopy levels (Figure 11E,F). Notably, the collagen fibrils exhibited a variety of orientations, often appearing cross-stitched or interwoven (Figure 11E). Additionally, the thickness of the collagen fibrils varied significantly (Figure 11F). Thickened fibrils displayed a regular banding pattern, whereas thinner fibrils showed a smoothing of the banding.

## 3. Discussion

Due to the complexity and multifaceted nature of the disease, patients with OA experience a wide array of symptoms that negatively impact their quality of life, which is significantly influenced by various factors, including both physical conditions like pain and immobility and neuropsychiatric conditions.

An important, yet not fully understood, aspect of the disease pathogenesis is the role of local and potentially systemic inflammation. In contrast to inflammatory arthritides, osteoarthritis is characterized by low-grade inflammation [44]. It is necessary to know the exact contribution of various factors to the development of inflammation, especially its systemic segment, for better therapeutic intervention. Some evidence points to excess weight and adipose tissue as a source of inflammation in osteoarthritis [45,46], although S. Reza Jafarzadeh et al. (2020) [47] showed that decreased weight positively affects pain but not the extent of synovitis. Other studies show the impact of overweight and the positive influence of diet and exercise on OA symptoms [48,49,50]. Our study demonstrates a positive correlation between BMI and urinary TNF-α, but not with the synovitis score or immune cell infiltration. This suggests that fat tissue may have a more significant impact on systemic inflammation than it does on OA in our cohort. Kanthawang et al. found that obese and overweight individuals have more significant knee synovial inflammation and associated structural and cartilage compositional degeneration, according to data from the Osteoarthritis Initiative [46,51]. The controversy between the aforementioned results and the hierarchical clustering results in this study highlights the necessity of conducting more clinical trials to correctly identify OA patient subgroups based on radiological imaging, serological, and omics analysis data.

Although inflammation is significant, the pain remains the predominant symptom of OA and is the primary reason patients seek medical attention [52]. Despite its significance, effectively managing the pain syndrome remains challenging due to the unclear and multifaceted pain mechanisms involved in OA [53]. Other scientists have investigated a correlation between inflammation in OA and pain perception [54,55]. These studies have primarily relied on either synovitis imaging or blood sample analysis, which are non-specific and not well-suited for assessing the low-grade synovial inflammation characteristic of OA. Our study did not find a correlation between the WOMAC score, which includes pain assessment, and the synovitis score. However, a cluster analysis revealed differences between OA subgroups with high-grade synovial inflammation. The pathogenesis of pain in OA involves various mechanical and molecular pathways, resulting in nociceptive, inflammatory, and neuropathic pain variants, which can alter mental and psychiatric status [13].

Patients with OA are susceptible to having depressive symptoms and anxiety, which adversely affect a patient’s well-being. Multiple studies in different populations have shown that up to 20% of patients with OA experience moderate to severe depression [55,56,57,58]. Other authors have shown that patients affected by depression are more likely to be female and have a higher BMI [46,59]. This statement is in line with our observation, which appeared during the investigation of OA patient subgroups when conducting a cluster analysis. Furthermore, consistent with other studies [34,60], our study showed that females with OA had a significantly higher risk of experiencing depressive symptoms.

Another area impacting patients’ lives and well-being is the worsening of cognitive function in OA [61]. While OA synovial tissue has a lower inflammatory profile compared to rheumatoid arthritis, it still exhibits more inflammation than healthy controls [62], and many patients with advanced OA experience synovitis [33]. Interestingly, in our study, one-quarter of the patients showed moderate to high levels of histological synovial inflammation. Studies suggest that mental decline in OA patients might be linked to inflammation, and the use of nonsteroidal anti-inflammatory drugs could be beneficial in treating the condition [63]. We observed a correlation between the presence of inflammatory cells in synovial tissue—a direct indicator of synovial inflammation—and a decline in cognitive performance as measured by MoCA. This suggests that synovial inflammation may be linked to mental and cognitive health in some patient cohorts. Although we found a negative correlation between MoCA scores and age, which validates our cohort, we also identified additional factors that may influence MoCA scores. Specifically, we found correlations between inflammatory indices and cognitive decline, depression, and anxiety. Another point to consider in our study is that females have better MoCA results than males.

The patients recruited in this study presented with advanced end-stage OA, which was confirmed by high KL OA severity grades. Because of the later OA stage, we did not perform MRI tests on the patients, and we performed ultrasound only for a few patients in case of excluding inflammatory arthritides. Although we did not use MRI to assess the presence of synovitis as a fluid effusion in the joint cavity or analyze bone marrow lesions, we believe that histopathological analysis is a more precise tool for analyzing inflammation within the joint.

A growing body of evidence indicates the utility of analyzing urine metabolites and biomarkers for diagnostics and research purposes [64]. Urine analysis is often preferred due to its non-invasive nature and ease of collection. Urinary biomarkers provide a non-invasive and readily accessible method for evaluating an individual’s health and risk for various diseases, including OA. Some studies have already confirmed the relevance of urinary examinations for collagen degradation products and their association with the severity of OA [65]. Other studies have highlighted the usefulness of COMP as a promising diagnostic and prognostic indicator and as a marker of OA severity and treatment efficacy [66,67]. Earlier studies have shown that women with the highest baseline COMP levels had a 48% increased risk of developing painful radiographic knee OA; however, this association was not significant after adjusting for age and BMI [68]. Some evidence suggests that measuring COMP in biological fluids, including urine, has potential as a predictive marker for advanced OA in horses [37,69]. To the best of our knowledge, our study represents the first investigation conducted in humans. CTX-II and COMP are among the most validated OA biomarkers, having been tested in many studies [37]. However, results vary and sometimes contradict each other, which is likely due to differences in patient types, study designs, and statistical analyses [70]. Given our discovery of a correlation between CTX-II and MOCA, further examination in patients experiencing cartilage degeneration and progressive cognitive decline associated with OA is warranted. However, we did not find any correlations between X-ray results and the urinary ELISA-explored biomarker indices, possibly due to the lack of diverse grading in osteoarthritic joint damage, as all patients were in the end-stage preoperative phase of OA.

As previously mentioned, we found no correlation between ELISA-confirmed urinary inflammatory markers, such as TNF-α, and synovial inflammation. In turn, approaching the discussion of another crucial marker, TGF-β1, which is a complex cytokine, and considering its possible multifunctionality—reflected by multiple, and sometimes diverse, biological effects on fibroblast proliferation, extracellular matrix deposition, tissue remodeling and degradation, and pleiotropic cytokine action with both suppressive and inflammatory immune responses [71,72,73]—we found a correlation between ELISA-confirmed urinary TGF-β1 levels and the synovitis score in this study. The patients recruited in this study presented with advanced end-stage OA, which was confirmed by high KL OA severity grades. However, we found no correlations between X-ray and the urinary ELISA-explored indices of the biomarkers studied. Because of the later OA stage, we did not perform MRI tests on the patients, and we performed ultrasound only for a few patients in case of excluding inflammatory arthritides. Our study’s urinary ELISA assay-based biomarkers further reinforce the notion that biochemical changes associated with joint inflammation, tissue remodeling, and degradation are linked to systemic inflammatory responses that affect mental health.

Animal models show pathological collagen synthesis in early osteoarthritis, preceding even the histopathological changes [74]. Studies show controversial ultrastructural findings in osteoarthritic patients. Significant changes in osteoarthritic cartilage and synovium are fibril disorganization [75]. Others also show thinning of collagen fibers and changing to collagen I fibers [76,77,78]. Other studies showed collagen fibril thickening during the progression of OA [79,80]. Our study corresponds to those studies, although there is a difference. Thus, we showed a disorganized matrix with tumid collagen fibers, which have different morphologies along the entire length of the fiber. This may indicate both disruption in synthesis caused by various stimuli in fibroblasts and the activation of metalloproteinases like ADAMTS [81]. Conversely, scattered data across several patient populations can point to distinct patient characteristics, leading to different disease phenotypes that need more investigation.

The results of this study should be considered in light of certain limitations. One common limitation in many medical studies is the sample size of the cohort investigated. This study explored inflammation, pain, and cognitive dysfunction in a moderately-sized cohort of OA patients. However, we included both male and female subjects to assess any sex differences and excluded patients with chronic diseases that may cause pain and affect cognition. Additionally, the absence of a control group without OA or other inflammatory joint diseases is a limitation. However, this reflects the specific focus of our study on exploring the interconnections between inflammation, pain, and cognitive function exclusively in OA subjects. Another issue pertains to the use of cognitive tests. We applied tests consistent with general practitioner and rheumatologist practices, including the MoCA test and the PHQ-9 and GAD-7 questionnaires. More complex tests typically run in psychiatric clinics, and the application of modern imaging modalities that allow the detection of affected domains was beyond the scope of this investigation. Finally, there are limitations related to the precision in identifying damaged supportive tissue altered in OA and using molecular biomarkers to indicate changes in the extracellular matrix composition. According to the literature, CTX-II has been detected not only in damaged articular cartilage but also in the tidemark and calcified cartilage zones, and it may also appear in cases of bone degeneration [40,74,82].

## 4. Materials and Methods

### 4.1. Patients’ Characteristics and General Clinical and Laboratory Data

The cohort comprised fifty patients with advanced OA undergoing joint endoprosthetic surgery for the disease at the Riga East University Hospital Clinic “Gailezers”, who were enrolled between January 2020 and January 2024. The inclusion criteria were a primary diagnosis or previously established clinical and radiological diagnoses of OA, aged between 36 and 78 years, and no objective or subjective evidence of any other joint inflammatory disease. All subjects met the relevant American College of Rheumatology (ACR) criteria for the following affected joints: hip [83] and knee [84]. Both male and female subjects were recruited to assess any sex differences. Patients with multiple joint OA or other inflammatory rheumatological conditions were excluded from this study. Patients with chronic diseases that may affect cognition, such as type 1 or type 2 diabetes mellitus, uncontrolled hypertension, previously diagnosed myocardial infarction or severe coronary heart disease, stroke or other known neurocognitive disorders, previously diagnosed severe atherosclerosis, blood vessel stent procedures, chronic severe lung diseases like COPD or severe and/or uncontrolled asthma, or a history of narcotic use, were excluded from this study. Patients with other anatomical localizations of OA, such as in the hand or shoulder, were also excluded from this study. Clinical data included information on disease duration, course, and clinical features at presentation, as well as details of the treatment received. Several laboratory parameters, including complete blood count, hemoglobin, and C-reactive protein (CRP), were examined. Ultrasound (US) examination was used to confirm synovial hypertrophy, synovitis, suprapatellar joint effusion, cartilage degradation, and osteophytes, while X-ray examination confirmed key radiographic features, such as joint space narrowing, the presence of subchondral cysts, sclerosis, and osteophytosis. The radiographic severity of joint OA was scored using an overall severity score (Kellgren/Lawrence (KL) grade) of the most severely affected joint, ranging from 0 to 4, based on weight-bearing fixed-flexion joint radiographs. Additionally, CT was used to assess bone OA changes, and an MRI examination evaluated bone marrow changes, cartilage loss, and soft-tissue joint structures. This study was approved by the Ethical Committee of Riga Stradins University (Decisions No. 6-1/01/62/2020, 30 January 2020, No. 4/671/2023, 27 November 2023) and conducted in accordance with the Declaration of Helsinki. Informed consent was obtained from all study participants.

### 4.2. Self-Assessment and Cognition Survey for OA Patients

Based on the recommendations of previous studies [85], we aimed to exclude any medical factors that could negatively affect cognitive or mental test results. Therefore, when performing cognitive testing, we ensured that the patient was relatively relaxed and trusted the examiner, who was a trained, experienced clinician, and other stress factors were minimized. The face-to-face survey was conducted by experienced, certified practitioners familiar with the research purpose and data collection procedures. Fifty OA patients were evaluated for physical functioning, mental health, and cognitive function before surgery, without preoperative pharmacotherapy influence. The same assessments were conducted post-survey in a quiet environment. Patients completed questionnaires under the supervision of a certified rheumatologist.

The Western Ontario and McMaster Universities Osteoarthritis Index (WOMAC) [86] and visual analog scale (VAS) measurements were utilized to assess the condition of OA patients, including pain, stiffness, and physical functioning of the joints. Total WOMAC scores were calculated as the unweighted sums of all 24 elements, ranging from 0 to 96. Next, we categorized WOMAC patient groups into mild (<60), moderate (60–80), and high-grade (>81) symptoms [87].

The Montreal Cognitive Assessment (MoCA) test was employed to gauge the severity of cognitive function impairment in OA subjects. Various cognitive domains were evaluated, including visuospatial/executive, naming, memory, attention, language, abstraction, delayed recall, and orientation (to time and place). A cut-off of 26 points out of 30 was utilized to indicate a mild, whereas a cut-off of 18 points was used to indicate a moderate decline in cognitive function.

The levels of depression and anxiety among OA patients were assessed using the Patient Health Questionnaire-9 (PHQ-9) [88] and the General Anxiety Disorder-7 (GAD-7) [89] questionnaires. We utilized validated Latvian- and Russian-language versions of the PHQ-9 [90] and GAD-7 [91]. Depression scores were categorized from “0” (not at all) to “3” (nearly every day), with a maximum possible value of 21. Total scores of 5, 10, 15, and 20, representing cut-off points for mild, moderate, moderately severe, and severe depression, respectively, were used.

Similarly, GAD-7 scores ranged from 0 to 21, with scores of 0–4 indicating minimal anxiety, scores of 5–9 indicating mild anxiety, scores of 10–14 indicating moderate anxiety, and scores greater than 15 indicating severe anxiety.

### 4.3. Conventional Light Microscopy to Assess the Histopathology of the Synovial Membrane

Joint replacement surgery was performed by a certified orthopedic surgeon on recruited subjects who met the criteria for surgical treatment, and synovial membrane tissue samples (*n* = 50) were collected from every OA patient undergoing joint replacement surgery. Histological sections measuring 4–5 μm in thickness were prepared from tissue samples fixed in 10% formalin and embedded in paraffin (FFPE). These sections were then mounted on SuperFrost Plus slides (Menzel GmbH, Braunschweig, Germany) for subsequent histopathological assessment. The images of interest were captured using a Glissando Slide Scanner (Objective Imaging Ltd., Cambridge, UK).

### 4.4. Histopathological Scoring of Synovitis Using Krenn and Morawietz Classification

To characterize synovitis, we utilized the grading system developed by Krenn and Morawietz [92]. Routinely stained slides (H&E) were employed, and the identified lesions in the synovial membrane were evaluated. The histopathological features assessed and scored included cellular hyperplasia of the lining layer, the cellular density of the sub-lining layer, and the presence of inflammatory infiltration, which was graded as follows: 0 for absent, 1 for mild, 2 for moderate, and 3 for strong. The total score obtained determined synovitis’s severity: 0–1 indicated no synovitis, 2–4 indicated low-grade synovitis, and 5–9 indicated high-grade synovitis. Given that OA typically manifests with chronic low-grade inflammation, the ranking of inflammatory alterations was further explored separately to better distinguish patients with inflammation more severe than the expected low-grade inflammation; the alterations were categorized into two groups: low inflammation grade and a higher-grade inflammation, which was termed moderate to strong inflammation.

### 4.5. Urine Sample Collection, Processing, and Measurements of CTX-II, COMP, TNF-α, and TGF-β1 Using Enzyme-Linked Immunosorbent Assay

Morning urine samples (minimum 5 mL per person) were collected from twenty-one OA patients who agreed to provide samples using 15 mL sterile tubes. These samples were left at room temperature for 30 min before being centrifuged at 3000 rpm for 10 min to isolate the supernatant, which was transferred into 200 μL microcentrifuge tubes and stored at −80 °C until analysis. An enzyme-linked immunosorbent assay (ELISA) was used to quantify CTX-II, COMP, TNF-α, and TGF-β1 urinary levels. Urinary CTX-II (Cloud-Clone Corp, CEA686Hu), COMP (Cloud-Clone Corp, SEB197Hu), TNFα (Thermo Fisher Scientific, BMS223-4), and TGFβ1 (Biorbyt, orb50103) levels were determined using commercially available ELISA kits following the manufacturer protocols. Urine samples were diluted as follows: CTX-II (1:2), COMP (1:3), TNFα (1:2), and TGFβ1 (undiluted). Sandwich ELISA kits were used for COMP, TNFα, and TGFβ1, with plates precoated with target-specific capture antibodies incubated with diluted samples and standards, followed by target-specific biotin-conjugated detection antibodies, avidin-conjugated horseradish peroxidase, substrate, and stop solutions, with thorough plate washing after each step. Absorbance was measured immediately at 450 nm. For CTX-II, a competitive inhibition ELISA kit was used. Plates precoated with target-specific capture antibodies were incubated with diluted samples, standards, and biotin-labeled CTX-II, followed by target-specific detection antibody, substrate, and stop solutions, with washing between steps. Absorbance was measured immediately at 450 nm. Absorbance measurements and result calculations were performed using the Varioskan™ LUX multimode microplate reader with SkanIt software (Thermo Scientific, Singapore, Singapore; version 7.0).

### 4.6. Assessment of Ultrastructural Synovial Membrane Alterations and Collagen Synthesis Using Transmission Electron Microscopy (TEM)

Since pro-inflammatory regulators released by synovial fibroblasts play a key role in the development of clinical symptoms of OA, such as inflammatory pain, and drive the inflammatory immune response in these cells, they have become focal points for exploring new comprehensive treatment strategies for OA [93]. To better explore the cellular characteristics of these cells, we used transmission electron microscopy. Another motivation for this application was the need to better study the peculiarities of synovial fibrosis, which has been shown to be closely associated with joint pain [94].

The joint tissue (synovial membrane and cartilage) samples were collected during endoprosthetic and joint replacement surgery. They were then cut into 1 mm^3^ tissue blocks and further processed for TEM. Following routine laboratory protocols, the samples were fixed in 2.5% glutaraldehyde in 0.1 M phosphate buffer (pH 7.2) and postfixed in 1% osmium tetroxide, dehydrated, and embedded in epoxy resin (Carl Roth 8623.1, 8639.1). Semithin sections 1 µm thick were cut with a PowerTome PCZ ultramicrotome (Boeckeler Instruments, Inc., Tucson, AZ, USA), collected on glass slides, and stained with 1% toluidine blue for analysis under a light microscope. Ultrathin sections 50 nm thick were cut with the same ultramicrotome, collected on Formvar-coated 200-mesh copper grids, and stained with uranyl acetate and lead citrate. Finally, the sections were examined with a JEM 1011 transmission electron microscope (JEOL, Akishima, Tokyo, Japan) at magnifications ranging from 3000× to 30,000×.

### 4.7. Statistical Data Analysis

A statistical analysis was conducted, and graphs were created using JMP Pro v17 (SAS, Cary, NC, USA) and GraphPad Prism v9.0 (GraphPad Software, San Diego, CA, USA), and JASP v0.18.3 [95]. Quantitative data were presented as medians with a 95% confidence interval, providing a range of the data. The Kolmogorov–Smirnov test was employed to assess the normality of the collected numerical data. Nonparametric tests were used to compare medians between different groups if the data were not normally distributed. The two-tailed Mann–Whitney U test was applied for comparisons between two groups, and Spearman’s correlations were utilized to explore relationships between values. Correlation strength was interpreted as follows: 0.2 to 0.4—weak, 0.4 to 0.7—moderate, and 0.7 to 0.9—strong. A significance level of *p* < 0.05 was considered statistically significant. Correlation matrices and clustering were used to provide a clear overview of associations among clinical data, self-reported tests, and synovial inflammation. Furthermore, hierarchical clustering was employed to identify patterns within the collected data of the study participants, assessing similarities and differences. Alluvial plots were generated using JAMOVI software v2.4.8 to visually represent associations across the categorical dimensions of the variables.

## 5. Conclusions

This study elucidates the intricate relationship among inflammation, pain, and cognitive decline in OA patients; however, further research in this field is needed to unravel the complexities of OA pain, leading to better-targeted therapies and improved patient outcomes. Our findings highlight a significant correlation between inflammatory markers, such as TNF-α and TGF-β1, and cognitive function, with elevated synovitis scores linked to poorer cognitive outcomes. Additionally, we demonstrate that urinary biomarkers, specifically CTX-II and COMP, may serve as valuable non-invasive indicators of cartilage degradation and overall disease progression in OA patients. Overall, these results underscore the necessity of considering the neuropsychiatric impact of OA in patient management.

## Data Availability

Data are available from the corresponding author upon reasonable request, preferably by e-mail: mihails.tarasovs@rsu.lv.
